# Visual light perceptions caused by medical linear accelerator: Findings of machine-learning algorithms in a prospective questionnaire-based case–control study

**DOI:** 10.1371/journal.pone.0247597

**Published:** 2021-02-25

**Authors:** Chao-Yang Kuo, Cheng-Chun Lee, Yuh-Lin Lee, Shueh-Chun Liou, Jia-Cheng Lee, Emily Chia-Yu Su, Yi-Wei Chen

**Affiliations:** 1 Graduate Institute of Biomedical Informatics, College of Medical Science and Technology, Taipei Medical University, Taipei, Taiwan; 2 Radiotherapy Division, Department of Oncology, Taipei Veterans General Hospital, Taipei, Taiwan; 3 Department of Medical Imaging and Radiological Technology, Yuanpei University of Medical Technology, Hsinchu, Taiwan; 4 Clinical Big Data Research Center, Taipei Medical University Hospital, Taipei, Taiwan; Dartmouth College Geisel School of Medicine, UNITED STATES

## Abstract

This study aimed to investigate the possible incidence of visual light perceptions (VLPs) during radiation therapy (RT). We analyzed whether VLPs could be affected by differences in the radiation energy, prescription doses, age, sex, or RT locations, and whether all VLPs were caused by radiation. From November 2016 to August 2018, a total of 101 patients who underwent head-and-neck or brain RT were screened. After receiving RT, questionnaires were completed, and the subjects were interviewed. Random forests (RF), a tree-based machine learning algorithm, and logistic regression (LR) analyses were compared by the area under the curve (AUC), and the algorithm that achieved the highest AUC was selected. The dataset sample was based on treatment with non-human units, and a total of 293 treatment fields from 78 patients were analyzed. VLPs were detected only in 122 of the 293 exposure portals (40.16%). The dataset was randomly divided into 80% and 20% as the training set and test set, respectively. In the test set, RF achieved an AUC of 0.888, whereas LR achieved an AUC of 0.773. In this study, the retina fraction dose was the most important continuous variable and had a positive effect on VLP. Age was the most important categorical variable. In conclusion, the visual light perception phenomenon by the human body during RT is induced by radiation rather than being a self-suggested hallucination or induced by phosphenes.

## Introduction

There is a long history of research on radiation-induced visual light perceptions (VLPs). In 1895, after discovering the X-ray, Wilhelm Röntgen conducted an experiment to determine whether X-rays could generate phosphenes, and verified that X-rays could generate VLPs; however, he could not definitively confirm that phosphenes were induced by X-ray irradiation [1]. In 1962, Garcia *et al*. conducted an early laboratory experiment on rats, and subsequently used electroencephalography to study changes in the brain activity of rats in response to radiation. The authors found that sleeping animals experienced changes due to radiation within less than 1 second of exposure [2,3].

For the first time during a space flight, the astronauts of the 1969 Apollo Mission reported experiencing VLPs, such as light flashes. The main contributory factors possibly included cosmic rays in outer space that directly stimulated the retinal photoreceptors or VLPs induction in the occipital cortex of the brain [4]. In 2016, Chuard *et al*. discovered that the phosphene mechanisms of eye proton therapy (PT) might be related to those of space radiation. The authors concluded that phosphenes could be produced by two mechanisms: indirect interaction of Cherenkov light in the eye due to scattering and direct simulation, or the excitation of excessive free radicals in nerve fibers located in the posterior part of the eye, close to the retina [5].

Thus, VLPs were mostly experienced by either astronauts [6] or patients who received radiation therapy (RT) for brain tumors [7]. The results of correlation studies have indicated that the retina is responsible for visual signal transmissions to multiple areas of the brain. There is clear evidence that long-range photon irradiation of neuron structures can lead to changes in VLPs [8].

Machine learning algorithms incorporate statistics to identify patterns that could be used to make predictions in the dataset [9], and have increasingly been used to learn from big data to obtain more reliable predictions [10]. Breiman proposed random forests in 2001 [11]; since then, the random forest algorithm has been widely used in computational medicine and biology [12]. Random forests converge due to the Law of Large Numbers and do not generate overfitting without pruning in prediction. Random features and inputs often achieve satisfactory performance in classification. Many recent studies have incorporated random forests because of its unique advantage in handling complex datasets with small sample sizes and high-dimensionality. Therefore, random forests have become one of the most effective analytical methods because of its high predictive capacity [13–16]. Moreover, random forests have performed better than other methods in the prediction and evaluation of fluorodeoxyglucose-positron emission tomography [17,18]. Furthermore, in the prediction of patient-specific quality assurance for volumetric modulated arc therapy, random forests have higher sensitivity than the Poisson lasso model in clinical and technical validation [19].

This study was conducted with an aim to identify the main factor that induces VLPs in patients who receive radiation therapy. We compared logistic regression and random forest analyses to select the best-performing method for predicting the outcome in terms of the receiver operating characteristics (ROC) curve and to discuss the relationship between the outcome and causative factors as these algorithms fit the dataset to not only predict the outcome but also to ascertain how the main factors modulate the outcome.

## Materials and methods

### Study objects

**Fig 1:**
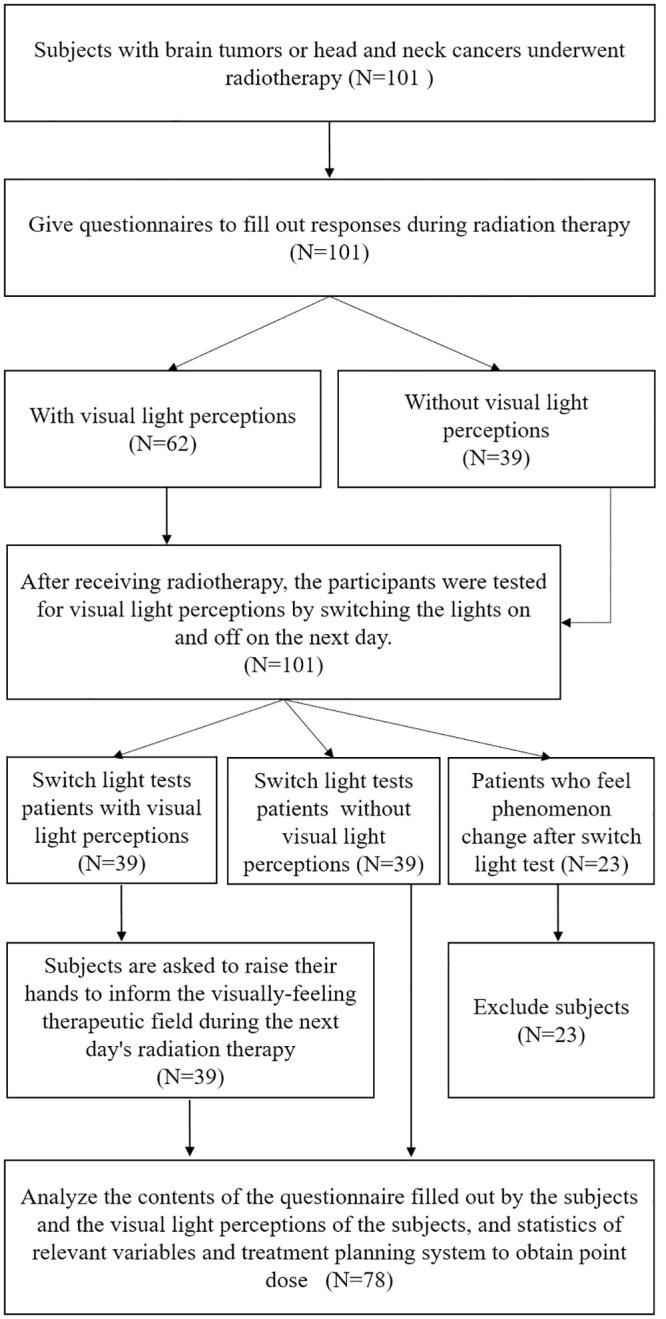
Flowchart and exclusion process.

This prospective cohort case-control study was approved by the Institutional Review Board of Taipei Veterans General Hospital (2016-09-025C). Between November 2016 and March 2017, we screened 101 patients for study participation, and 23 were subsequently excluded after failing the visual perception light switch test and the visual perception radiation field time point verification test. During the fractional treatment, after the completion of the questionnaire, the timepoint verification tests of light and VLPs were conducted with the consent of the subjects. The test was performed by delivering the treatment with all lights turned off in the treatment room and asking the patient to identify whether they had experienced VLPs. The purpose of this test was to exclude VLPs caused by the treatment room light or the field light during irradiation. The research workflow and patient disposition schema are shown in Fig 1.

The 78 (49 male, 29 female) participants included 48 patients who were receiving brain irradiation and 30 head-and-neck RT patients. The brain and head-and-neck RT patients were further subdivided into two groups according to their dose schemes and radiation fields as: an experimental group with VLPs (n = 39) and control group without VLPs (n = 39). Subsequently, 6/10 MV photon beams were delivered to patients in the experimental group using A-brand or B-brand dual-energy medical linear accelerators, and the dose rate (DR) was limited to 400–1600 MU/min. Subjects were asked to complete the study questionnaires and interviews after they had received RT. The treatment plan of the corresponding patient in the treatment planning system (TPS) was then reviewed according to the results of the questionnaire.

Patients who were eligible for study participation included men or women aged 20–85 years, with various levels of education, who were fully conscious, had no mental disorders, could communicate with medical staff and fill out questionnaires, demonstrated normal visual function, and were irradiated in either the brain or the head-and-neck area.

Based on the questionnaire results, the E-TPS and the V contouring software was used to identify the area that induced VLPs during irradiation by reviewing and analyzing the patients’ output dose, DR, beam angle, intra-fractional dynamic radiation field, and isodose lines, which delineate the visual path and irradiation field of the brain. After the completion of the questionnaire, information was collected in the TPS to analyze the corresponding dose distributions.

### Switch light test

The timepoint for the switch light test, with the participant’s consent, was specified as the completion of the questionnaire in the study process flowchart. The duration for which the lights are turned on will be determined in conjunction with each participant’s treatment plan, mainly before and after the initiation of RT, whereas the lights will only be turned off during the actual RT of the subjects. Therefore, comparing the response to the questionnaire that was previously filled out by each subject with the VLPs response that was recorded during the treatment/the sub-treatment was used to check whether each participant’s VLPs would change as a result of the treatment. White and yellow bulbs were used in the room, the lamp seat was distributed on the ceiling of the treatment room where the machine is installed, and the indoor bulbs (brightness 27 Watts) are situated 2–4 m away from the participants.

From this test, we discovered that the subjects could be further assigned to three categories. The first category included patients who experienced VLPs regardless of whether the lights were on or off. The second category included patients who did not experience any VLPs regardless of whether the lights were on or off. The third category included patients whose VLPs disappeared once the lights were turned off. Consequently, participants in the third category were excluded from the analysis to ensure that they would not be a source of bias in the data collection. In addition, the field timepoint verification test with VLPs was performed by turning the treatment room light on and off during photon irradiation and asking the patients to notify the staff by raising their hands when they experienced any VLPs in the treatment field.

### Point-dose method

**Fig 2:**
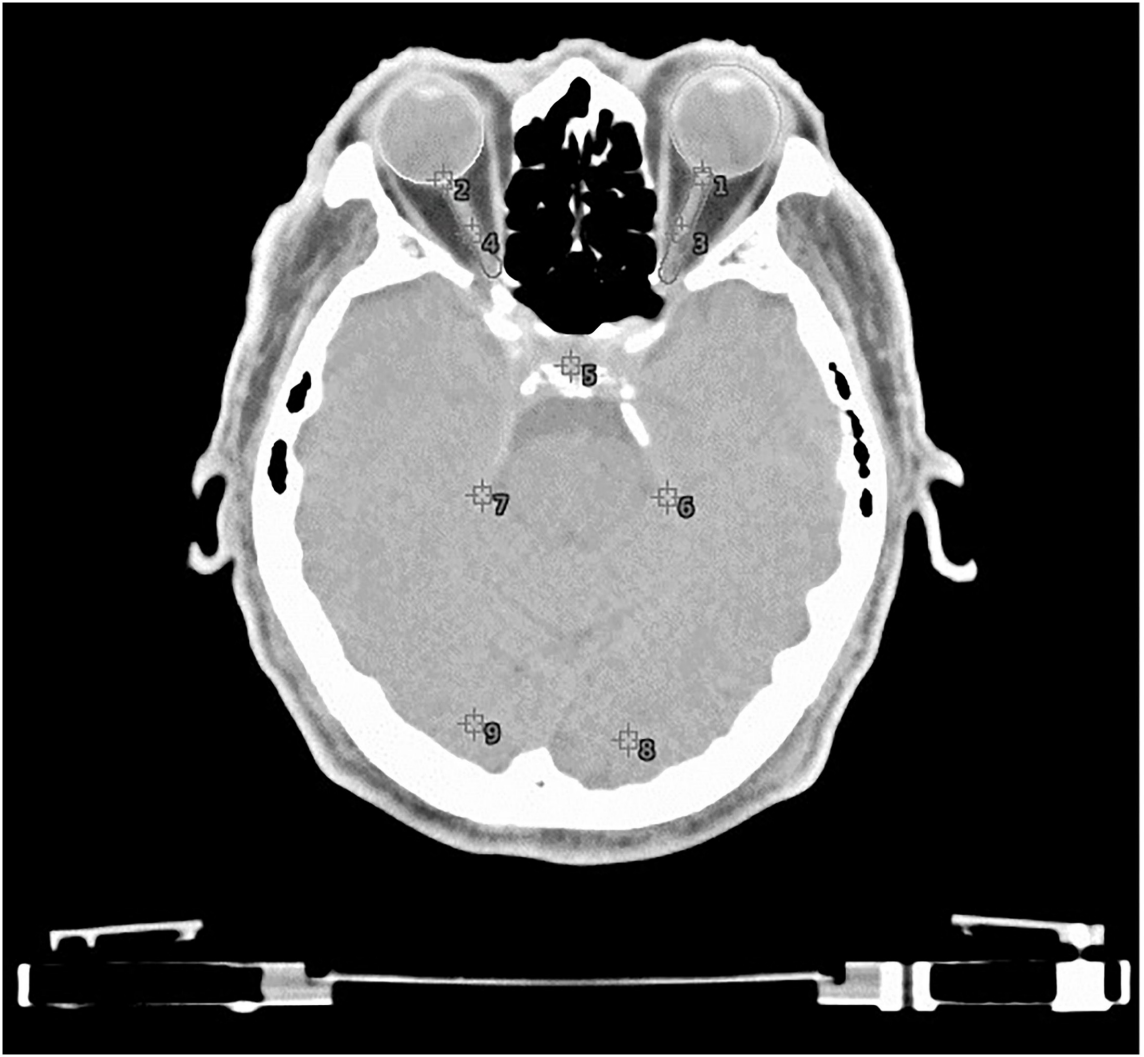
The point-dose method to analyze the fraction dose of the computed tomography axial view.

In the TPS, we adopted the point-dose method to analyze the fraction dose (FD) that was delivered to certain parts of the optic path to facilitate a comparison on specific points at different areas of the optic path (Fig 2). To determine the location of these points, magnetic resonance images were compared with those obtained from computed tomography scanning. Then, with assistance from senior specialists, the points were located according to their relative positions to the bones and soft tissues. The points used for comparison were placed at various locations, including the retina, optic nerves, optic chiasm, lateral geniculate nucleus, and visual cortex. The FD of the subjects varied individually.

### Statistical analyses

Statistical analyses were conducted by using SAS 9.4 (SAS Institute Inc., Cary, NC, USA.) and RStudio version 1.2.5001 (2009–2019 RStudio, Inc.). The frequencies and percentages were calculated for categorical variables, and the means and standard deviations were computed for continuous variables. Between-group comparisons of the baseline characteristics of participants with and without VLPs were analyzed by the Student’s *t*-test for continuous variables and the chi-square test for categorical variables, and differences were considered statistically significant if the *p*-value was less than 0.05.

### Machine learning algorithms

Logistic regression (LR) has a similar model as linear regression, and the effect estimates and *p*-values can be obtained directly from the regression output. The difference between them is that linear regression analyzes continuous dependent variables, and LR is used for binary or multinomial dependent variables and it estimates the probability of occurrence of the interested outcome [20,21].

In the random forest model, we incorporated the random forest package developed by Breiman and Culter in the R environment [11]. The random forest algorithm estimates a specific variable importance by observing how much the prediction error increases when out-of-bag (OOB) data for a specific variable are permuted, with all other variables remaining the same [11]. The varImpPlot in the package is a function which generates a dot chart that is measured by the random forest model [22]. After comparing the importance of variables, we applied partialPlot function to generate partial dependence plots which depict the marginal effects of variables on the class probability for classification, and they provide insights on variable influences for black box machine learning algorithms [23] and show relative propensities in logit distributions of the class probability [22]. Positive values on the y-axis indicate that the values of independent variables are more likely to be in the positive class. In contrast, negative values are less likely to be in the positive class. Obviously, zero indicates the absence of an average influence on class probability.

### Evaluation measures

We used four measures, including accuracy, sensitivity, specificity, and area under the curve (AUC), to evaluate the prediction performance. Accuracy, sensitivity, and specificity are defined as follows:

$$Accuracy=\frac{TP+TN}{TP+TN+FP+FN}$$

$$Sensitivity=\frac{TP}{TP+FN}$$

$$Specificity=\frac{TN}{TN+FP}$$

where TP, TN, FP, and FN denote the numbers of true positives, true negatives, false positives, and false negatives, respectively [24].

Sensitivities and specificities are shown in the ROC curve diagram wherein the *x*-axis represents 1 − specificity and *y*-axis denotes sensitivity. The curves with higher sensitivity and specificity are close to the upper left corner of the diagram, and those with less sensitivity and specificity are close to the diagonal line. Previous studies have suggested that the AUC is a better indicator for comparing and measuring the performance of classification algorithms [25]. This is because the AUC avoids the assumed subjectivity in threshold selection when the continuous probability is converted into binary positive–negative dependent variables and summarizes the model performance under all possible thresholds [26].

## Results

A total of 293 samples filled by 78 participants were collected, and 41.64% of the participants had experienced VLPs. For categorical variables, we used chi-square tests to examine whether two variables were independent. As shown in Table 1, energy was the only variable which was significantly correlated with VLPs, whereas age and sex were not associated with VLPs. We used the Student’s *t*-test to examine whether the means showed significant differences between continuous variables and VLPs. The means of all variables, except FD, showed significant differences.

**Table 1:** Baseline characteristics of continuous and categorical variables.

		With Visual Light Perception (n = 122)		Without Visual Light Perception (n = 171)		*p*-value
**Continuous Variable, Mean, SD (cGy)**		**Mean**	**SD**	**Mean**	**SD**	
Fraction dose		216.00	37.67	224.20	41.48	0.082
Retina fraction dose		30.99	38.01	10.02	20.23	<0.001***
Optic nerve fraction dose		161.60	73.96	120.80	86.23	<0.001***
Optic chiasma fraction dose		42.95	47.57	24.70	43.46	<0.001***
Lateral geniculate nucleus fraction dose		40.75	48.29	23.12	43.03	0.001**
Visual cortex fraction dose		37.34	50.33	20.44	43.09	0.003**
**Categorical Variable, N, %**		**N**	**%**	**N**	**%**	
Energy	6 MV	86	29.35	141	48.12	0.016*
	10 MV	36	12.29	30	10.24	
Age, years	<65	103	35.15	131	44.71	0.100
	≥65	19	6.48	40	13.65	
Sex	Male	80	27.30	125	42.66	0.167
	Female	42	14.33	46	15.70	
**Color for Visual Perception**		**N**	**%**	**N**	**%**	
Total		122	100.00	-	-	
White		49	40.16	-	-	
More than one color		40	32.79	-	-	
Blue		13	10.66	-	-	
Yellow		9	7.38	-	-	
Orange		7	5.74	-	-	
Red		2	1.64	-	-	
Purple		2	1.64	-	-	

*p<0.05,

**p<0.01,

***p<0.001.

SD, standard deviation; cGy, centiGray; N, Number.

^a^ The radiation dose is the dose for a single course of treatment at the location of the lesion.

**Fig 3:**
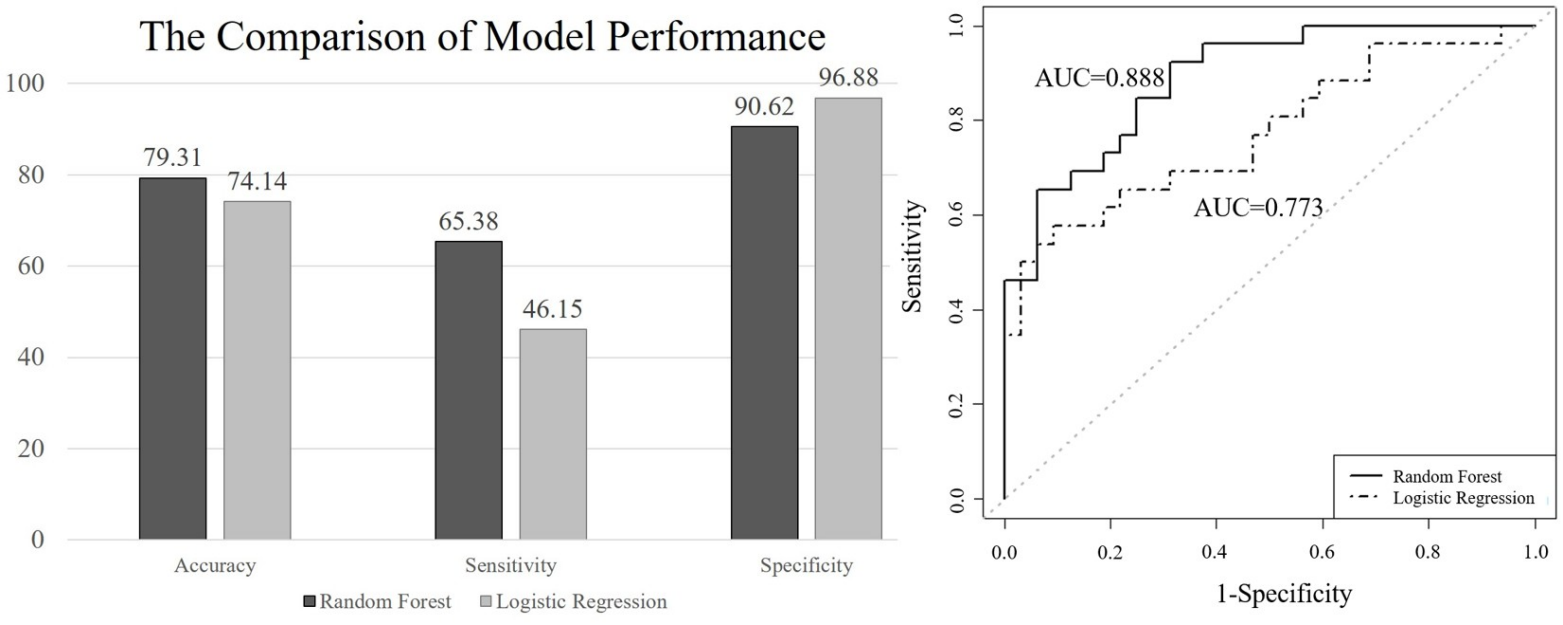
Predictive performance in the test set between random forests and logistic regression.

Furthermore, we compared random forests to LR and selected the best model according to the comparison of AUCs. To avoid overfitting of predictive performance, it is common in machine learning to randomly divide the original dataset into a training set and a test set, wherein only the training set is incorporated to develop a prediction model, and the test set is used to evaluate the real predictive performance. We randomly divided the original dataset into 80% as the training dataset, with 235 observations, and 20% as the test set, with 58 observations. The random forests built 5,000 trees and randomly sampled three variables at each split in each tree. In the results, random forests outperformed LR in both training and test sets in terms of accuracy, sensitivity, and AUC, although the specificity was lower than in the LR model (Fig 3). In the test set, random forests achieve 0.888 of AUC, compared with 0.773 by LR. Therefore, we chose random forests as the best model.

**Fig 4:**
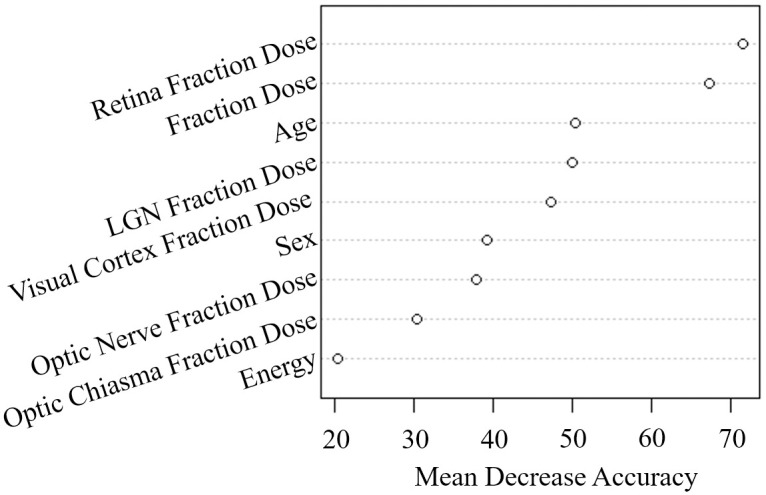
Importance of variables based on random forests.

To further depict the importance of variables, Fig 4 ranks the variables according to their mean decrease accuracy (MDA), which is a measure of the difference of classification that is presented as the average over all trees in the forest and calculates the out-of-bag error rate between an original dataset, where the variable is included, and a randomly permuted dataset [27–29]. Interestingly, retina FD and FD showed a higher MDA than other variables, which suggests that these two variables are important factors affecting VLPs. Among the categorical variables, age was the most important variable (Fig 4).

**Fig 5:**
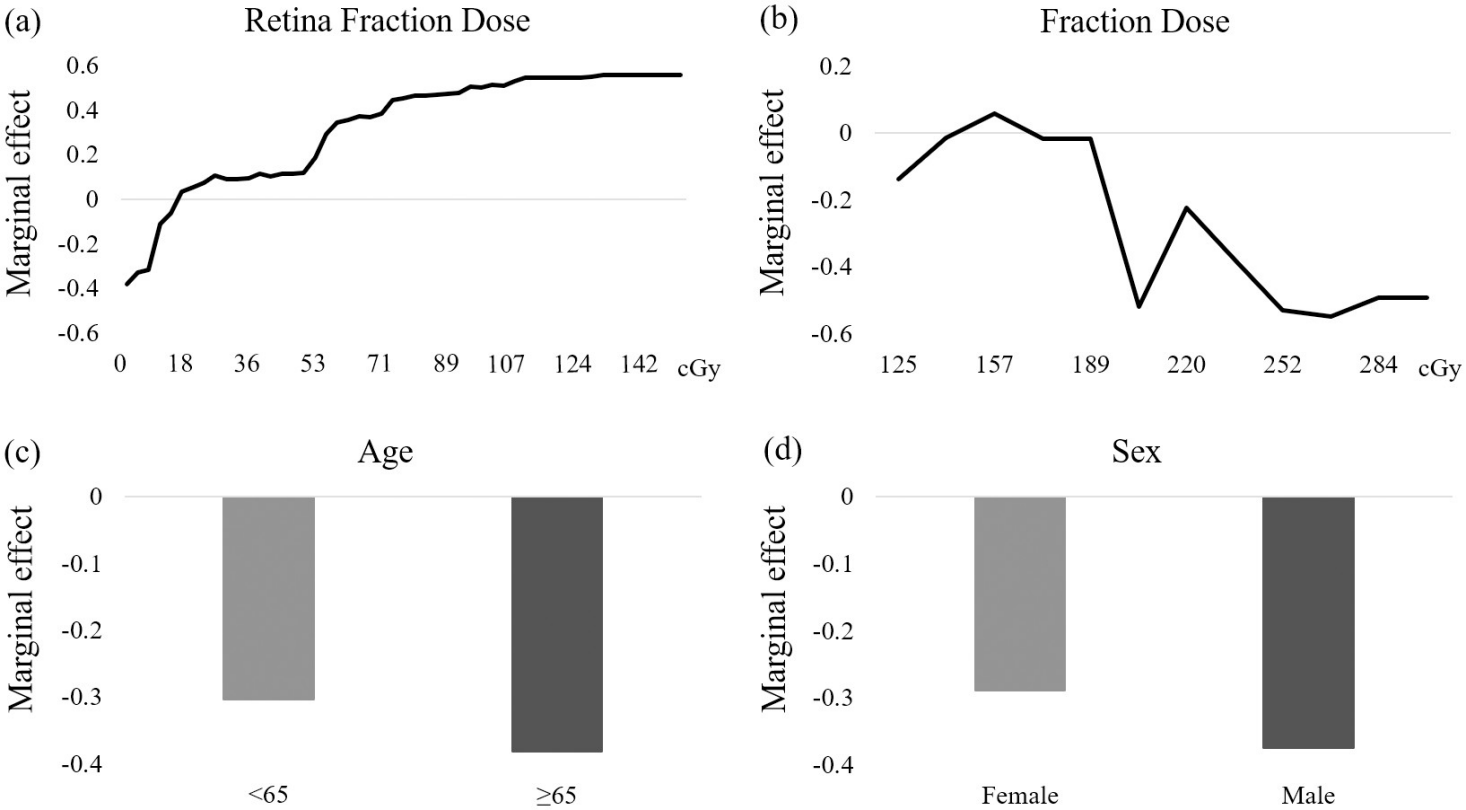
Relationships between variables and visual light perceptions depicted by partial dependence plots. Continuous variables are presented as line plots, and categorical variables are presented as bar plots. The probability of VLPs induced by (a)retina FD, (b)FD, (c)age, and (d)sex.

Fig 5 illustrates the partial dependence plots that depict the relationships between visual perception and predictors. All variables are labeled on the *x*-axis of individual plot, respectively. The *y*-axis shows the changes in the fraction of vote for probability of VLPs in each variable. The continuous variables are retina FD and FD, and they exhibit significant nonlinear relationships. As shown in Fig 5a, the retina FD curve has a positive effect on VLPs, wherein higher retina FD causes a higher propensity of visual perception especially when the retina FD exceeds 18 cGy. Fig 5b demonstrates that the FD shows a flat slope and a negative effect to visual perception, and that higher FD reduces the propensity of VLPs as it exceeds 157 cGy. In the categorical variables of age, when compared with patients younger than 65 years, patients who were older than 65 years had less propensity in VLPs (Fig 5c). In terms of sex (Fig 5d), male patients had less propensity, compared with female patients, for treatment-induced VLPs.

## Discussion

This study focused on whether the VLPs experienced by patients receiving RT was caused by phosphenes or was an actual result of radiation. The basis of this research theory is to construct the visual light perception phenomenon during the RT of the brain and head and neck. This study analyzed whether VLPs could be affected by different radiation energy, prescription doses, age, sex, or RT locations, and whether all VLPs were caused by radiation. Phosphenes can be considered to be a kind of light hallucination, which is characterized by the ability to sense light when no visible light enters the eyes. The normal VLPs of the human body is produced by light conversion. In addition, phosphenes can be produced in other ways without light stimulation; for example, they also appear in the research records of transcranial magnetic stimulation [30].

The direct priming mechanism of the retina is the same as that of visible light [31]. In our study, the retina FD shows a positive effect to the propensity of VLPs (Fig 5a). This means that as the age increases, the incidence of VLPs decreases. According to the figures of FD and FD of the retina, the position of irradiation is the key point to induce VLPs in subjects during treatment.

In our subject group, categorical variables of patients younger than 65 years had a higher incidence of visual perception than those from patients older than 65 years (Fig 5c). The questionnaire data from subjects who experienced VLPs during the study indicated that the radiation-induced light or VLPs was reproducible. Then, rather than occurring only in a single treatment, VLPs was repeatedly induced in every treatment field of every fraction.

The primary beams used in this study are high-energy X-ray photon beams, the characteristics of which are different from those of the proton, heavy-ion, and electron beams. From our research questionnaire and statistics (Table 1), it seems that the phenomenon of VLPs in patients during treatment does not require a high dose or energy to be induced. In comparison with the VLPs caused by PT or heavy ion therapy mentioned in the literature, radiation has higher energy than conventional RT and can be derived from the statistics of this study. Both 6 and 10 MV photons induced VLPs, which suggested this result. Several studies showed that more Cherenkov photons are produced for beams of higher energies [32], and VLPs are attributable to Cherenkov radiation [33]. However, our hypothesis states that irradiation energy may have to combine with other variables (e.g., retina or optic nerve) to induce VLPs. Therefore, it is considered that energy is not the only key to induce VLPs; rather, the most important cause may be the location of the visual pathway. This finding contradicts what previous studies exploring Cherenkov light generation have concluded, thus more patients may be needed for further assessment on this issue.

Radiation was induced in the eyes mostly by the indirect interaction between radioactive decay and nucleus [5]. In the course of RT, Cherenkov radiation (CR) energy was produced in the eyes and may be helpful in inducing VLPs [33]. The CR produced by eyeballs may be the origin of VLPs in patients with head and neck cancer treated by RT [34]. During PT, 60% of the subjects experienced VLPs, among which 74% saw a blue light. In contrast, only 41.64% of the subjects in this study experienced VLPs, among which only 10.74% observed a blue light. There was a convincing evidence that the color of the VLPs really depends on the generation mechanism; therefore, most of the blue light of CR occurs in the eyes [5]. This result indicates that the high-energy X-rays produced by medical linear accelerators create different visual perception incidences than that of PT. Alternatively, heavy-ion therapy uses an energy range of 80–400 MeV/n, whereas the medical linear accelerators used in this study can only deliver energies of 6 and 10 MV. However, despite the substantial energy difference, most heavy-ion therapy subjects reported observing a white light, with only 10% of the subjects reporting a yellow light. This observation is highly consistent with our results that most subjects experienced a white light (40.5% of the subjects). Therefore, we can conclude that photon therapy and heavy-ion therapy induced similar VLPs of white light in patients [35].

A previous study suggested that patients would experience VLPs regardless of whether the eyes were open or closed, which is consistent with our questionnaire results. Furthermore, our results indicate that the retina FD is significantly correlated with the incidence of VLPs, which is contradictory to the conclusion in the literature that most of the VLPs happen in the optic path areas beyond the retina [8,31].

Finally, the limitations of this study mainly include the shape or the type of light, the time coordinate axis of induced VLPs during treatment, and other variables that were not evaluated in this study. The CR observed in radiotherapy is affected by predictable factors in patient imaging, such as radiation beam characteristics, tissue thickness, entrance/exit geometry, curved surface effect, curvature, and imaging angle. Experiments have found that changes in CR signal have varying degrees of influence on tissues [32]. However, all possible spatial and time delivery rates used in pencil beam scanning (PBS) proton therapy [36], as well as heavy particle therapy modulated with 3D scanning [37], are indeed significantly different from traditional RT techniques and dose rates [38]. Moreover, the proton pencil beam dose rate affects the patients’ image. In the future, we could try to evaluate the correlation between the patients’ image and VLPs and try to compare the modulation of VLPs under different RTs. Therefore, we hope to track whether subjects with VLPs have visual impairment or pathological changes, and the identification of associations between VLPs with chemotherapy or surgery could be an interesting aspect to work on. However, the sample size of this study was relatively small for machine learning because there was a limited number of patients with brain tumor and head-and-neck cancer in this short study period. A large-sample analysis with more samples will be undertaken after the latest certificate of the Institutional Review Board (IRB) is approved. Moreover, in subsequent studies, we hope that individualized assessment of RGB-coded palettes would more accurately determine the colors that they see. In addition, future studies should include more variables, including dose rate, into the model.

## Conclusion

Random forest is a good and stable methodology in binary classification. In the comparison of two models measured by accuracy and AUC, random forests outperform LR in terms of the predictive ability. In this study, the most important continuous variable was the retina FD, and the most important categorical variable was age. The VLPs phenomenon in the human body during RT was indeed induced by radiation rather than being a self-suggested hallucination or induced by phosphenes. Radiation-induced VLPs often demonstrates complicated conditions and warrants special attention. Whether radiation-induced VLPs can lead to side effects, such as visual impairment, requires further investigation.

## Supporting information

S1 Dataset (CSV)

S1 File (DOCX)
