# Correction: Visual light perceptions caused by medical linear accelerator: Findings of machine-learning algorithms in a prospective questionnaire-based case–control study

**DOI:** 10.1371/journal.pone.0252634

**Published:** 2021-05-27

**Authors:** Chao-Yang Kuo, Cheng-Chun Lee, Yuh-Lin Lee, Shueh-Chun Liou, Jia-Cheng Lee, Emily Chia-Yu Su, Yi-Wei Chen

In the Funding section, the funding source the Higher Education Sprout Project by Ministry of Education (MOE) was omitted. The correct funding statement is: “The research and publication charge were supported by the Ministry of Science and Technology (MOST) in Taiwan (grant number MOST109-2221-E-038-018) and the Higher Education Sprout Project by Ministry of Education (MOE) in Taiwan (grant number DP2-110-21121-01-A-13) to Emily Chia-Yu Su. The funders had no role in study design, data collection and analysis, decision to publish, or preparation of the manuscript. There was no additional external funding received for this study.”
