# Supplementary material for: Visual light perceptions caused by medical linear accelerator: Findings of machine-learning algorithms in a prospective questionnaire-based case–control study
Source: PLoS One. 2021 Feb 25;16(2):e0247597. doi: 10.1371/journal.pone.0247597 (PMC7906346; doi:10.1371/journal.pone.0247597)
Supplement: S1 File — (DOCX) [file pone.0247597.s002.docx]

**Visual light perceptions Questionnaire**

1. Do you feel flashes or bright lights during radiotherapy? (Yes, No)
2. During the treatment, how did your eyes look when you feel this visual perceptions?

(Open eyes, close eyes, both)

1. Is the visual perceptions you feel is flashes or continuous light? (Flash, bright light)
2. Does this visual perceptions source also appear in the CT positioning before treatment?

(Yes, No)

1. Please try to describe the color of light? (Black, white, red, orange, yellow, green, blue, indigo, purple)
2. Is the light only visible when it is irradiated (ie when the machine beeps)?

(Yes, No)

1. How do you feel the visual perceptions brightness (the higher the score, the stronger the brightness) level? (Weak, mild, moderate, strong, harsh)
2. Do you feel visual perceptions every time of you receive treatment? (Yes, No)
3. Excuse me, what area did you treat this time? (Brain, head and neck, chest and abdomen, others)
4. What type of visual perceptions appears? (Point, line, surface, three-dimensional, irregular, fixed, mobile)
5. Please try to describe the directionality of light? (Front and rear, left and right, up and down directions)
6. The bright spots of light are divided into five areas. What areas appear on the field of view position map? (Please draw on the map, for example: fixed-point light source or moving light or distribution)

13. Did you wear a mask during this course of treatment? (Yes, no)

14. Have you ever received chemotherapy before? (Yes, no)

15. Have you ever underwent surgery before received radiotherapy? (Yes, no)

16. May I ask where you have undergone surgery? (None, brain, neck, others)

17. Do you have color blindness? (Yes, no, not clear)

18. During the radiotherapy, did you have the above visual experience when you were told to take the verification film? (Yes, no)
